# Characterization of *Plasmodium falciparum* structure in Nigeria with malaria SNPs barcode

**DOI:** 10.1186/s12936-018-2623-8

**Published:** 2018-12-17

**Authors:** Bolajoko E. Bankole, Adeyemi T. Kayode, Iguosadolo O. Nosamiefan, Philomena Eromon, Mary L. Baniecki, Rachel F. Daniels, Elizabeth J. Hamilton, Katelyn Durfee, Bronwyn MacInnis, Henrietta Okafor, Akintunde Sowunmi, Sarah K. Volkman, Pardis Sabeti, Dyann Wirth, Christian T. Happi, Onikepe A. Folarin

**Affiliations:** 1 African Centre of Excellence for Genomics of Infectious Diseases, Redeemer’s University, Ede, Osun State Nigeria; 2 Department of Biological Sciences, Redeemer’s University, Ede, Osun State Nigeria; 3 Broad Institute of MIT and Harvard, Cambridge, MA USA; 4 Harvard T.H. Chan School of Public Health, Boston, MA USA; 5 Department of Paediatrics, Institute of Child Health, University of Nigeria Teaching Hospital, Enugu, Enugu State Nigeria; 6 Department of Pharmacology and Therapeutics, College of Basic Medical Sciences, University of Ibadan, Ibadan, Oyo State Nigeria; 7 Simmons College, Boston, MA USA

**Keywords:** Malaria, *Plasmodium falciparum*, SNP, Barcode, HRM, Genotype, Nigeria, West Africa

## Abstract

**Background:**

*Plasmodium falciparum* malaria remains a major health challenge in Nigeria despite the global decline of its incidence and mortality rates. Although significant progress has been made in preventing the transmission of *P. falciparum* and controlling the spread of the infection, there is much to be done in the area of proper monitoring, surveillance of the parasite, investigating the population dynamics and drug resistance profiling of the parasite as these are important to its eventual eradication. Polymorphic loci of *msp1*, *msp2* and/or *glurp* genes or microsatellites have been traditionally used to characterize *P. falciparum* population structure in various parts of Nigeria. The lack of standardization in the interpretation of results, as well as the inability of these methods to distinguish closely related parasites, remains a limitation of these techniques. Conversely, the recently developed 24 single nucleotide polymorphism (SNP)-based molecular barcode assay has the possibility of differentiating between closely related parasites and offer additional information in determining the population diversity of *P. falciparum* within and between parasite populations. This study is therefore aimed at defining the population diversity of *P. falciparum* in and between two localities in Nigeria using the SNPs barcode technique.

**Methods:**

The 24-SNP high-resolution melt (HRM) barcode assay and *msp2* genotyping was used to investigate both intra and inter population diversity of the parasite population in two urban cities of Nigeria.

**Results:**

Based on SNP barcode analysis, polygenomic malaria infections were observed in 17.9% and 13.5% of population from Enugu and Ibadan, respectively, while *msp2* analyses showed 21% and 19.4% polygenomic infections in Enugu and Ibadan, respectively. Low levels of genetic diversity (π) of 0.328 and 0.318 were observed in Enugu and Ibadan parasite populations, respectively, while the F_ST_ value of 0.02 (p = 0.055) was obtained when the genetic divergence of both populations was considered.

**Conclusions:**

The 24-SNP barcode assay was effective in analysing *P. falciparum* population diversity. This study also showed that *P. falciparum* populations in Enugu and Ibadan had a degree of intra-population diversity, but very low divergence between the population. A low degree of polygenomic infections were also observed in the two parasite populations unlike previous years. This maybe as a result of the effect of artemisinin-based combination therapy (ACT), long-lasting insecticide-treated nets (LLITNs) and intermittent preventive treatments in the study populations.

## Background

Malaria caused by *Plasmodium falciparum* is still a major health challenge in Nigeria despite the global decline of its incidence and mortality rate [1]. Malaria is estimated to kill between 81,000–150,000 people yearly in Nigeria [1]. Even with the adoption of artemisinin-based combination therapy (ACT) as the first-line treatment in malaria, the use of intermittent preventive measures (IPT) for pregnant women, and the use of long-lasting insecticide-treated nets (LLIN), there has not been the expected reduction in the incidence, prevalence, or mortality of malaria in Nigeria [1]. As such, Nigeria alone is still contributing to 30% of the total world malaria burden. This situation underscores the need to develop and implement new tools to monitor parasite population dynamics and diversity in order to measure the impact and success of different intervention measures across Nigeria [2].

The use of molecular markers such as the merozoite surface protein 1 (*msp1*), merozoite surface protein 2 (*msp2*) and glutamine rich protein (*glurp*) to establish the population features of *P. falciparum* in various parts of Nigeria is well documented [3–5]. The lack of a standard and/ reference for the interpretation of results across borders is a major disadvantage to the use of these techniques [6]. Microsatellites, which are short tandem repeats (STRs) in the genome of *P. falciparum,* have also been used in characterizing *P. falciparum* field isolates with good success [5]. The possibility of microsatellites rapidly evolving is a major challenge for use as genotyping technique [7].

The malaria barcode [8] is a combination of SNPs that express a unique pattern of variation on the *P. falciparum* genome sequence, combined with high-resolution melt (HRM) analysis [9] is more accurate in determining the population diversity of *P. falciparum* and has the ability to differentiate between closely related parasites. SNP genotyping approaches have also been used to evaluate interventions, pursue malaria elimination and monitor malaria transmission [10, 11]. HRM analysis is a simple polymerase chain reaction (PCR) performed under slightly different or modified conditions in the presence of a specific dye developed for the detection of DNA sequence variants [9]. HRM is based on amplicon melting in which the SNPs allele is determined by the melting curve, i.e., monitoring the dissociation rate of the DNA with a dye and plotting the progress as a melt curve [12]. The melt curve is determined by factors such as heterozygosity, GC content and sequence length. The *P. falciparum* barcode also uses separate probes for each assay, allowing better peak differentiation for more accurate SNPs genotyping, which makes it different from other HRM applications. The gradual increase in temperature during the melting stages of the HRM allows for the detection of slight genetic differences like SNPs. A major advantage the HRM SNP barcode has over the use of molecular polymorphic markers such as *msp1*, *msp2* and *glurp* is the ability to detect changes in population structure such as decline or increase in transmission rate or relatedness of isolates based on identity by descent. However, this technique is not as effective in differentiating reinfection/recrudescence as in the polymorphic markers i.e., *msp1* and *msp2*. Also, HRM is relatively easy to use, simple, flexible, low cost, non-destructive in nature, very sensitive and specific [8].

The 24-SNPs barcode assay can be used to detect changes in parasite population features by tracking parasite genotypes over time and through routes of vector and human migration [10]. For instance, it has been applied to show the emergence of highly related *P. falciparum* parasites in Senegal [10], interrogate the impact of intervention strategies in Zambia and Zimbabwe [11] and investigate outbreaks from clonal expansion of *P. falciparum* in Panama [9]. However, no such study has been conducted or reported in Nigeria. This study therefore seeks to leverage on this novel genotyping technique for the first time in Nigeria to study *P. falciparum* obtained from blood samples of children with uncomplicated malaria, to have an insight to the population structure of the parasite.

## Methods

### Study site

**Fig. 1:**
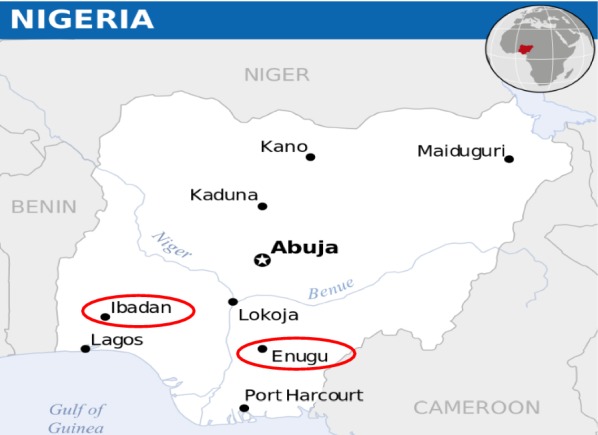
Map of Nigeria showing the study sites where *Plasmodium falciparum* samples were collected from children with uncomplicated malaria infection

Samples used in this study were obtained from two major urban cities, Ibadan and Enugu in Nigeria (Fig. 1). Ibadan is in the southwestern part of Nigeria (7.3775° N, 3.9470° E), where malaria is hyperendemic and transmission takes place all year-round [13]. The intense transmission of malaria in Ibadan makes it clinically difficult to distinguish between recrudescence and reinfection after treatment. Enugu on the other hand, is in the southeastern part of Nigeria (6.4584° N, 7.5464° E). There is a high malaria transmission all year-round, with an average malaria incident rate of 15% during dry season and 35% during wet season [14].

### Sample collection

This study was part of a larger drug efficacy study investigating the effect of artemether-lumefantrine (AL) and artesunate-amodiaquine (AA) in the treatment of uncomplicated malaria infection. Two drops of finger prick blood was blotted on 3 mm Whatmann filter paper (Whatmann International Limited, Maidstone, UK) before treatment and during follow up on days 3, 7, 14, 21 and 28. However, DNA was extracted from dried blood spot on filter papers obtained before treatment only and was used for this parasite diversity study.

### DNA extraction

DNA was extracted from dried blood impregnated filter paper using Qiagen DNA extraction kit and according to the manufacturer’s protocol. Briefly, one-quarter of the dried blood spot was used for extraction and DNA content was eluted in a final volume of 60 μl with buffer AE (Qiagen DNA extraction kit).

### Allelic typing of *msp2* gene

The polymorphic block 3 of the *msp2* gene was amplified by nested PCR following protocols described by Happi and others [4]. PCR reactions were carried out in a final volume of 25 µl using family specific primers and Illustra™ PuReTaq Ready-To-Go PCR Beads (GE Healthcare UK Limited, Little Chalfont Buckinghamshire, Lot 9618045). Polymorphisms on *msp2* alone were used for characterization of parasite population structure in patients’ samples because previous studies from Nigeria have shown that it is the most reliable marker for studying *P. falciparum* population diversity in the country [4, 15–18]. Two microlitres (2 µl) of the secondary amplification products were resolved by electrophoresis on a 2% Agarose gel and sized against 100 bp molecular weight marker (New England Bio labs, Beverly, MA).

### 24-SNPs molecular barcode

We initially quantified parasite genomic DNA by qPCR against a standard curve of known concentrations. All quantifications were done in triplicates and *P. falciparum *HB3 clone genomic DNA (MR4 stock number MRA-155) was used as control. A pre-amplification step was then performed on samples with parasite DNA concentration lower than 0.1 ng/µl [19]. All samples (both pre-amplified and not pre-amplified) were further subjected to genotyping analysis using the 24 SNP-based parasite molecular barcode assay. Barcoding assay was carried out as described by Daniels and others [8].

### Genotype determination

**Fig. 2:**
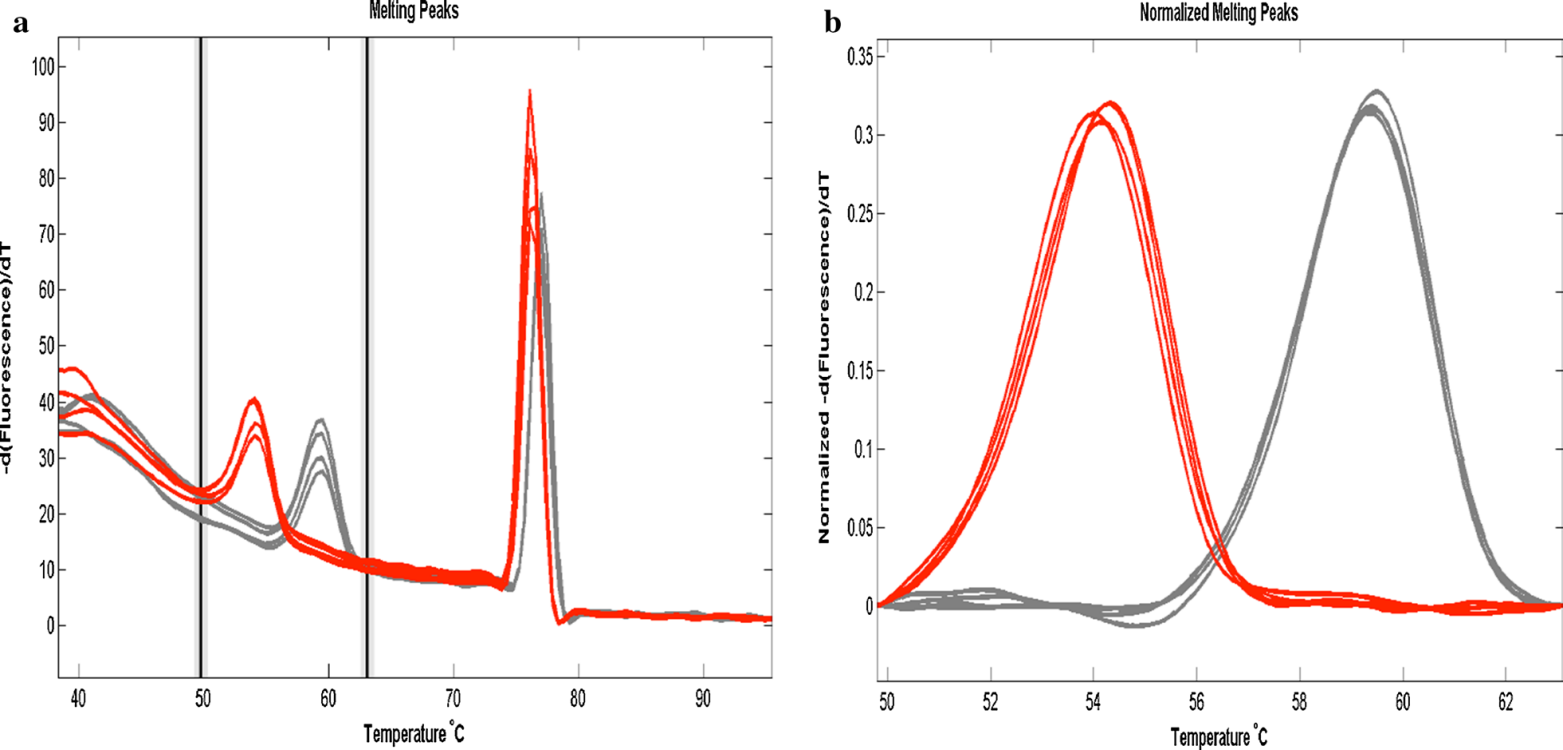
Melting curves for SNP 12 in the 24-SNPs barcode analysis of *Plasmodium falciparum* isolates obtained from children from Enugu and Ibadan showing reference allele (red) and alternate allele (Grey). **a** Raw plot of the melting curve for SNP 12. **b** Normalized plot of the melting curve for SNP 12

The HRM assay was run on the Roche LightCycler 480 Real-Time PCR System using the manufacturer’s settings and the cycling conditions as stated by Daniels and others [8]. The LightCycler 480 Software (release 1.5.1) was also used to analyse the results and make genotyping calls. The reference and alternate alleles of the controls were used to define the SNP of the sample for each of the 24 assays. Samples were called using the melt curve genotyping workflow, in which the software automatically compare melting peaks of the samples to those of the controls (Fig. 2). However, the data was also manually changed in case of any missed, incorrect, or ambiguous genotyping calls. A sample was designated to have mixed (N) infection if peaks that matched two of the control genotypes at a specific locus were observed. A sample was designated to be negative (X) if it showed no discernable peaks or whose peaks did not match those of any of the controls. These genotyping calls were compiled to give the complete 24 SNP barcode for each sample, which was then subjected to further analysis.

### Statistical analysis

#### Data cleaning

Prior to analysis of the raw barcode data, proper clean up measures were taken to ensure that only high quality data were considered for further analysis. Two (2) major parameters were used to clean the data, these were: (i) only ATCG, N (mixed infection) or X (missing SNP) should be present in the barcode data (ii) and samples with barcodes containing more than 4 missing SNPs (X) were excluded from the analysis.

#### Complexity of infection

Polygenomic infections was established in the parasite population by examining the number of heterozygous SNPs (N) in each sample assayed. It is popular knowledge that the human blood-stage malaria parasites is haploid, therefore, a monogenomic infection should have only one allele at each SNP locus while a polygenomic infection is expected to carry multiple alleles. Therefore, in categorizing the parasite population as polygenomic, a minimum threshold value of at least two (2) heterozygous SNPs (N) was utilized as defined by Sisya and others [20]. This is because a single random SNP out of the 24 SNPs genotyped is occasionally wrongly scored as heterozygous even in well-characterized monogenomic infections. Thus, samples were categorized as monogenomic infections if without any or at most, one (1) heterozygous SNPs (N) in the barcode [20].

#### Minor allelic frequency

Minor allelic frequency (MAF) was computed according to methods earlier described by Baniecki et al. [12]. Briefly, MAF was calculated from allele counts for each SNP in each population. For each polymorphic genotype calls for both the reference and alternate alleles were calculated by designating each with a half contribution compared to monomorphic genotypes. The average MAF (AMAF) defined as the unweighted mean of the MAF values for both populations for each SNP was further determined.

#### Population diversity (Barcode, π)

The population diversity (π) was calculated as described by Baniecki et al. [12]. Barcode π is a measure of population diversity with values ranging from 0–1. Values closer to 1 suggest high population diversity [12]. Briefly, π was manually calculated as the mean of the pair-wise differences at assayed SNPs between all members of a population divided by the total number of assayed SNPs. In addition, for each monomorphic/polymorphic genotype, half the original value of the monomorphic/polymorphic mismatch was utilized [12].

#### Population divergence (fixation index: F_ST_)

The population divergence was measured by calculating the fixation index (F_ST_) for all pairs of populations. The online software, COIL (Broad Institute, USA) was used to determine the F_ST_ for both populations.

#### Principal component analysis (PCA)

Principal component analysis (PCA**)** was performed with the online program, ClustVis (https://biit.cs.ut.ee/clustvis/) on each of the parasite populations (Ibadan or Enugu) separately as well with both populations together.

## Results

### Data cleaning

A total of 100 samples, (50 each from Ibadan and Enugu) were run on HRM to achieve a full 24 SNP barcode. Coverage for 23 out of the 24 barcode assays were observed, as one was faulty, i.e., all samples and controls assayed for that SNP were negative. Omitting this, the overall data still included a rather high (25%) negative (X) genotyping calls. Therefore, data cleaning was essential for obtaining useful data for population diversity analysis. Overall, 37 and 28 samples from Ibadan and Enugu, respectively, produced barcodes with sufficient data quality and were considered for further statistical analyses, giving us a total of 65 samples.

### Population diversity and divergence

A total of 100 samples were tested using the *msp2* genotyping technique, from which 74 samples were successfully analysed. Analysis showed that 19.4% (7 of 36) and 21% (8 of 38) of the samples obtained from Ibadan and Enugu, respectively were polygenomic. The complexity of infection (COI) using *msp2* polymorphic marker were 0.73 and 0.52 in Enugu and Ibadan, respectively. Based on analysed SNP barcode data, 17.9% (5 of 28) and 13.5% (5 of 37) of samples from Enugu and Ibadan, respectively were polygenomic.

### Minor allelic frequency (MAF)

**Fig. 3:**
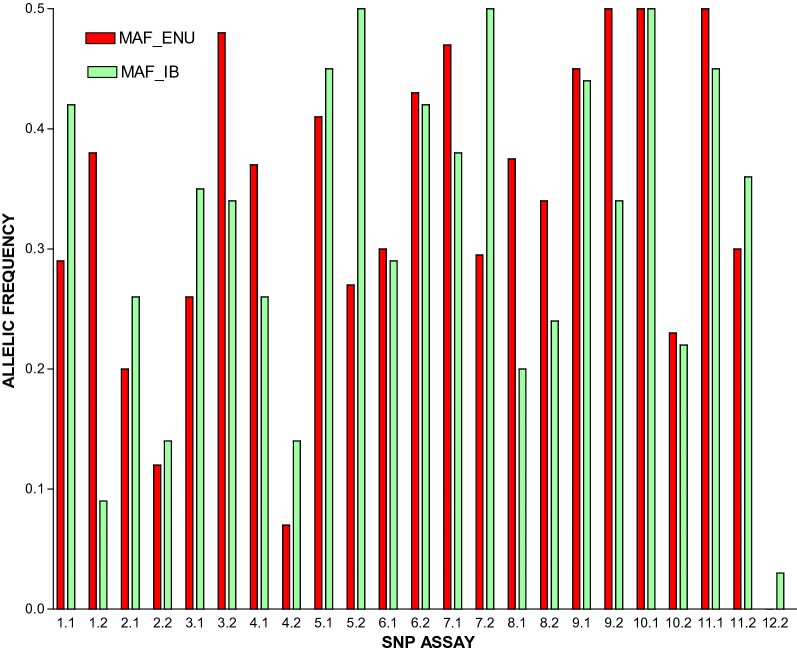
Minor allele frequencies (MAF) of *Plasmodium falciparum* isolates obtained from Children in Enugu and Ibadan

The minor allelic frequency (MAF) values for samples obtained from both populations showed that these populations are highly diverse. In both populations, only two SNPs had MAF values lower than 0.1: Enugu: assay 8 and 24, (Table 1A), Ibadan: assay 2 and 24 (Table 1B). While the populations showed high degree of intra-population diversity, the MAF values of the two populations compared to one another showed that the inter-population diversity was relatively low (Fig. 3).

**Table 1:**
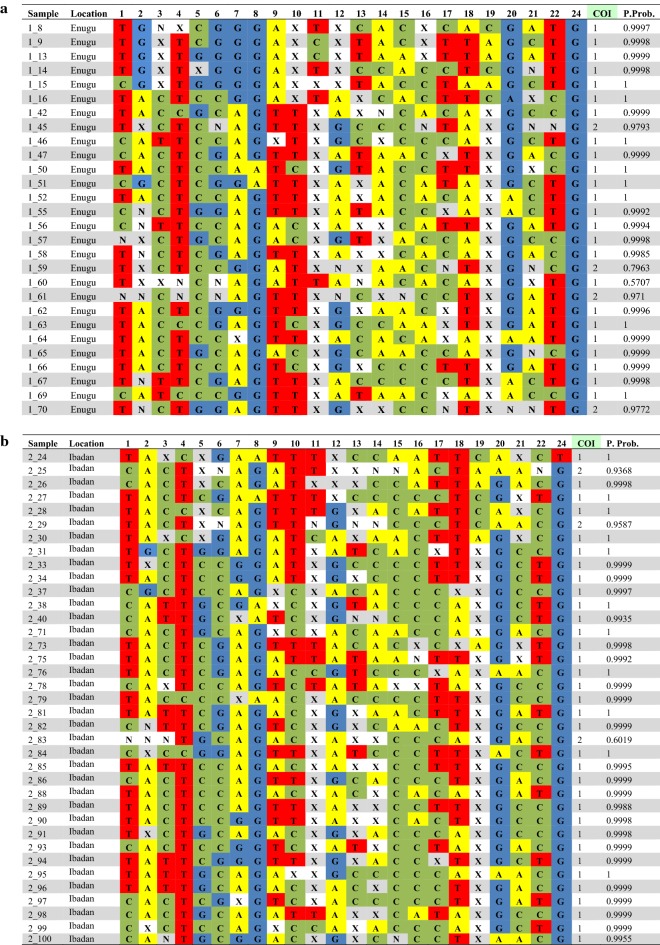
24-SNP Barcodes for 65 *Plasmodium falciparum* isolates obtained from children with uncomplicated malaria in Enugu and Ibadan

(a) 28 samples from Enugu and (b) 37 samples from Ibadan that passed data quality tests. Genotyping nucleotide calls of A, T, C and G were given to samples with one peak matching the corresponding peak for the reference or alternate allele of the previously sequenced controls (3D7, Dd2, MCamp, TM90). Samples with peaks matching both the reference and alternate alleles were marked as mixed infections (N). Samples with peaks matching neither of the control peaks were marked as Negative (X). Assay 23 was omitted from the data, as it failed to provide data for any of the samples assayed

### Population diversity (barcode π)

The intra population diversity values for Enugu and Ibadan were 0.328 and 0.318, respectively. The calculated π statistic shows that these populations are slightly diverse.

### Population divergence (fixation index: F_ST_)

The population divergence (F_ST_) value of 0.02 (p = 0.0549) suggests that both populations are not significantly genetically diverse from each other. Thus, confirming the similarity between the MAF values of the two populations.

### Principal component analysis (PCA)

**Fig. 4:**
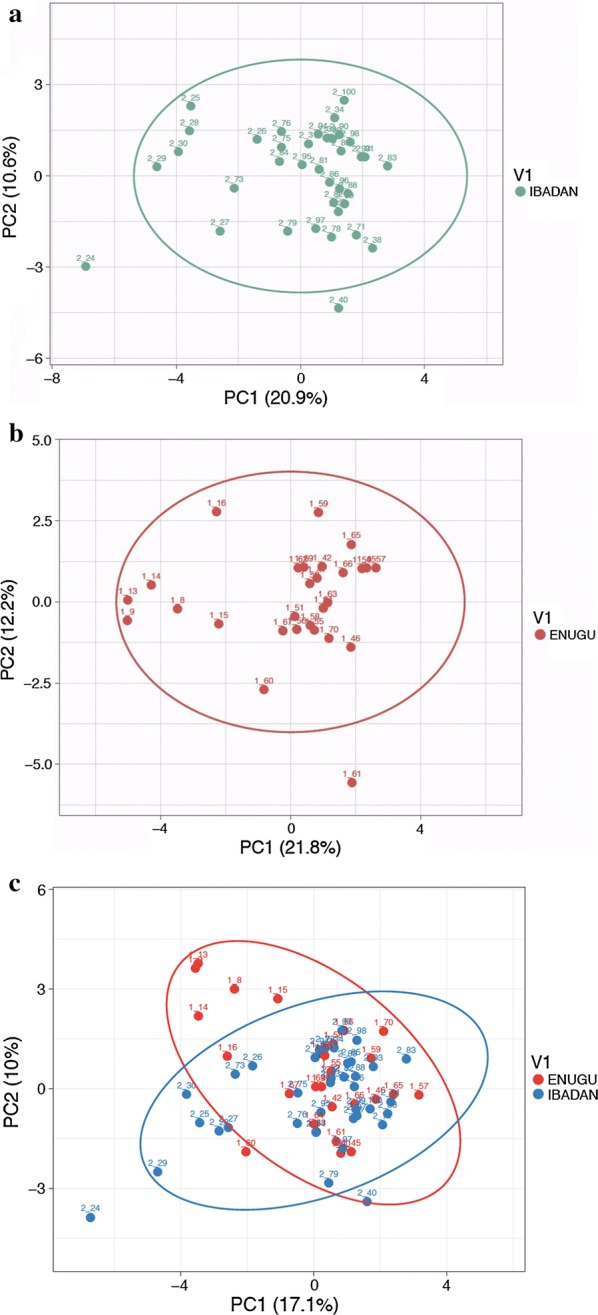
Principal component analysis (PCA) of *Plasmodium falciparum* isolates. **a** Principal component analysis of *P. falciparum* isolates obtained from Children in Enugu. **b** Principal component analysis of *P. falciparum* isolates obtained from Children in Ibadan. **c** Principal component analysis of *P. falciparum* isolates obtained from Children in Enugu and Ibadan combined

The online program, ClustVis was used to run the PCA of parasite populations from Enugu and Ibadan parasite separately as well as with all 65 samples together (those that were successfully barcoded). When the PCA was performed on each parasite population, the points showed more divergence with small clustering of the parasites in each study site (Fig. 4a, b) especially the Enugu population (Fig. 4a). The PCA plots further confirmed the similarity between the two populations, as the parasites appeared clustered around the same region when all samples were considered (Fig. 4c).

## Discussion

This study present the very first application of the 24-SNPs molecular barcode in effectively measuring inter and intra population diversity for *P. falciparum* samples in Nigeria. The HRM technique has been used to evaluate parasite population dynamics of *P. falciparum* in several parts of the world [8, 10, 20]. The HRM technique is a sensitive genotyping technique capable of identifying both known and novel polymorphisms, detect multiple genotypes that indicate mixed infections, distinguish between variants when multiple copies of a locus are present and easily deployable to field samples [21]. The knowledge of the distribution of complexity of infection (COI) across a patient population has been said to reveal information about disease transmission and epidemiology [22]. High complexity of infection has been found to be seasonal in Senegal and positively associated with the degree of parasitaemia in individual patients [23]. Over the years, the complexity of infection of *P. falciparum* in samples obtained from representative parts of the country has served as an indicator for transmission patterns of the parasite within the country and influenced various government control and intervention measures such as adoption of ACTs for malaria treatment, rapid diagnosis of all malaria cases, wider distribution of LLIN and quality monitoring of malaria cases to ensure the progressive control of malaria in Nigeria [24].

Previous molecular epidemiological studies showed high levels of polyclonal infections especially in malaria hyper endemic parts of the country, thus indicating high *P. falciparum* transmission within and between various parts of the country. For instance, Engelbrecht et al. [3] using the *msp2* genotyping technique, reported polyclonal infections at 80% from samples obtained in the Northern region of Nigeria. Previous studies in Ibadan using *msp2* reports 89–100% polygenomic infections in samples obtained prior to treatment [4, 25]. However, recent studies have presented the steady decline in clonality from polygenomic to monogenomic infections in Nigeria [5, 17, 26].

Observations in this study from the two parasite populations also revealed a similar trend of higher percent of monogenomic infections. The complexity of infection values obtained by *msp2* analysis in this study for both populations (Enugu: 0.73 and Ibadan: 0.52) suggest a low genetic diversity. This shift to monogenomic infections may be due to present use of ACT in the country, which has gametocide activity as well as capability to reduce parasite load rapidly. Thus, causing a reduction in the transmission rates and coupled with other malaria control efforts such as use of LLIN and IPT for children and pregnant women in the country.

HRM has been reported to be a better genotyping technique over the use of length polymorphic markers such as *msp2* [8], however Chi square analysis of prevalence of polygenomic infections in this study showed that there was no significant difference (Enugu, p = 0.46 and Ibadan p = 0.16) between both genotyping techniques. This may be due to small sample size used in the HRM analysis.

A major advantage the HRM technique has over other genotyping techniques such as *msp1*, *msp2*, and *glurp* is that the HRM is a more robust genotyping technique having the ability to resolve two closely related samples [8]. This is due to the wider SNPs coverage and also capability to determine other parameters that define population diversity.

Parasite population diversity parameters such as the AMAF and barcode π provide further insight to the nature of parasite diversity within Enugu and Ibadan. Baniecki et al. [12] had suggested that AMAF values higher than 0.1 represent a significant degree of diversity within that particular SNP of the barcode. In this study, AMAF values from both populations revealed a significant degree of SNP diversity (> 0.1) for all but one (assay 24: 0.02) SNPs of the 23 considered. The 24th SNP assay in the barcode showed 100% and 97% homogenicity for the reference allele in the Enugu and Ibadan parasite populations, respectively. This may be due to the absence of selective pressure for the alternate allele of this SNP in these 2 populations and regions. However, further barcoding assay of larger samples from these states and country in general may be required to confirm if the low occurrence of the alternate allele in the 24th SNP assay is a common trend. This may serve as a means of tracking transmission of parasite from other population with higher alternate allele occurrence. The barcode (π) was also determined to define the population diversity of the parasite in the two study sites. Results showed the π values 0.328 and 0.318 for Enugu and Ibadan respectively, suggest the existence of a low level of genetic diversity among the parasite in each of these populations. This may be due to a bottleneck caused as a result of selective pressures that eliminated other genetically diverse but susceptible *P. falciparum* clones originally present in these regions. The high prevalence of monoclonal infections observed in this study as well as other parameters determined in this study further supports evidence of the low genetic diversity of *P. falciparum* in these locations.

The population divergence of *P. falciparum* population between Ibadan and Enugu was investigated by computing population divergence features such as fixation index (F_ST_) and principal component analysis (PCA). When SNP barcode data of two populations are analysed the F_ST_ value ranges from 1 (high divergence) to 0 (no divergence) [27]. In this study, F_ST_ value of 0.02 between Ibadan and Enugu parasite populations was observed. This suggests that there is low genetic variation in circulating *P. falciparum* parasites of both populations. Although these two regions are over 500 km away from one another, they are by no means isolated thus encouraging circulation of same parasite population. The lack of genetic variance observed in this study could be due to constant and ease of travel between the two cities, similar epidemiological conditions in both areas and/or similar selective pressure from the various control and management measures within the country at large. A recent study has shown that great geographical distance, even at the continental level, does not necessarily result in high level of genetic divergence between populations [12]. The PCA of both populations further confirmed the genetic similarities of *P. falciparum* in Enugu and Ibadan. A similar clustering patterns were observed between the parasite populations (Fig. 4).

Based on the observations of low genetic divergence between Enugu and Ibadan populations as well as the low intra population diversity in each population, its suggested that HRM technique, although may be more expensive can be used to investigate the current trend of population diversity and divergence in other parts of the country. This would provide sufficient information about the composition of parasite populations across Nigeria and significantly influence controls measure and intervention for the spread of malaria.

In summary, results from this study showed that the two populations, Enugu and Ibadan, had a low degree of intra-population diversity, as samples analysed using both *msp2* and HRM techniques were to a large extent, monogenomic. This may suggest that the current malaria transmission rate in each of these regions is also low. The HRM method has proven to be a good genotyping tool and can reliably resolve single nucleotide changes. Furthermore, this 24-SNP barcode may be a useful tool for general surveillance, as continued cyclical screening of malaria-positive samples in regions across Nigeria will be invaluable for tracking major mutations and population-level trends. It can also provide data that will help in tracking malaria transmission and parasite evolution. Elucidating these parasites and population level differences using the 24-SNP barcode can inform clinicians’ treatment decisions, equipping them to recommend different sources of treatment with information beyond individual patient data and symptoms.

## Conclusion

This work demonstrates, that the 24-SNP HRM barcode can effectively be used to monitor *P. falciparum* population diversity and divergence in Nigeria. Future work is to utilize this assay to build on this characterisation of parasite populations in regions of varying endemicity across the nation. Also, reports from other studies has shown that this technique can be used to monitor changes in parasite population diversity and clonality over time, transmission patterns, and emerging drug resistance which were not considered in this study and could be included in future studies in Nigeria. By building a more complete snapshot of *P. falciparum* infection, policy makers, drug developers, and healthcare professionals can be empowered to take informed action to further the goal of malaria elimination in Nigeria.

## Abbreviations

ACT: artemisinin-based combination therapy
AMAF: Average Minor Allele Frequency
COI: complexity of infection
GLURP: glutamate rich protein
HRM: high resolution melt
IPT: intermittent preventive treatment
LLIN: long-lasting insecticide-treated net
MAF: minor allelic frequency
MSP: merozoite surface protein
PCA: principal component analysis
SNP: single nucleotide polymorphism
STR: short tandem repeats
